# Shear bond strength of eugenol- and non-eugenol–based endodontic sealers to gutta-percha and dentin: An *in vitro* study

**DOI:** 10.4103/0972-0707.43415

**Published:** 2008

**Authors:** Shibu Thomas Mathew, Mithra N Hegde

**Affiliations:** Department of Conservative Dentistry and Endodontics, A. B. Shetty Memorial Institute of Dental Sciences, Mangalore, India

**Keywords:** EDTA, instron, shear bond strength

## Abstract

**Aims:**

a) To evaluate the bond strength of eugenol- and non-eugenol-based endodontic sealers to gutta-percha and dentin and b) To make a comparative evaluation of the bond strength of sealers to gutta-percha and dentin.

**Material and Methods:**

Seventy-two specimens were used in this study, which was divided into two groups — group I consisting of 36 freshly extracted human molars with 2 mm coronal cut surface; and group II consisting of gutta-percha disks of 10 mm diameter and 2 mm thickness embedded in 36 plaster of Paris specimens. Group I was further divided into three subgroups: subgroup 1, subgroup 2, and subgroup 3, consisting of 12 teeth each; and group II was further divided into three subgroups: subgroup 1, subgroup 2, and subgroup 3, consisting of 12 plaster specimens each. Each subgroup consisted of two materials each, which were placed in 5 mm long sections of polyethylene tubing and were then placed on coronal 2 mm cut surface of human molars; likewise subgroup 1, subgroup 2, and subgroup 3 of group II consisted of two materials each, which were also placed in 5 mm long sections of polyethylene tubing and then placed on gutta-percha disks 10 mm in diameter and 2 mm in thickness, which were embedded in the plaster specimens. After the materials were set, the specimens were subjected to test for shearing bond strength using a universal testing machine (Instron).

**Statistical Analysis:**

Mann-Whitney *U* test.

**Results:**

Group I, i.e., zinc oxide eugenol sealers, showed more bond to gutta-percha than to dentin, in which subgroup 2b, endomethasone, showed the maximum bond. Group II, i.e., non-zinc oxide eugenol sealers, showed more bond to dentin than to gutta-percha, where the maximum bond was shown by subgroup 3b, EndoRez.

**Conclusion:**

Under the circumstances of this in *vitro* study, all the six endodontic sealers had significant differences in terms of shear bond strength to gutta-percha and dentin.

## INTRODUCTION

Successful root canal therapy requires a complete obturation of the root canal system with non-irritant biomaterials. It is known that the majority of endodontic failures have been caused by the incomplete sealing of root canal and restorative shortcomings, confirming the necessity of using materials capable of forming a fluid impervious seal between the root canal system and the peri-radicular tissues.[1] Strength of the bond of root canal sealers to gutta-percha and dentin seems to be the important property for maintaining the integrity of apical seal, and obtaining a fluid impervious seal, along with cleaning and shaping the root canal, one of the keys to achieve a long-term successful endodontic treatment.

A fluid impervious seal cannot be obtained without the use of a sealer, because gutta-percha does not spontaneously bond to dentin. Therefore, the ability of sealer cement to bond to tooth structure is of considerable importance.

The purpose of this study was to compare the shear bond strength of eugenol- and non-eugenol–based endodontic sealers to gutta-percha and dentin. Although models in different studies have permitted calculation of the strength of the bond of the sealer to gutta-percha, they could not disclose the exact value of the strength the bond to gutta-percha and dentin by each substrate. Furthermore, there are no studies that determine the bond strength of these newer endodontic sealers to both gutta-percha and dentin. This study was undertaken to test the hypothesis that there are differences in bond strength between eugenol and non-eugenol–based endodontic sealers to gutta-percha and dentin.

## MATERIAL AND METHODS

**Figure 1:**
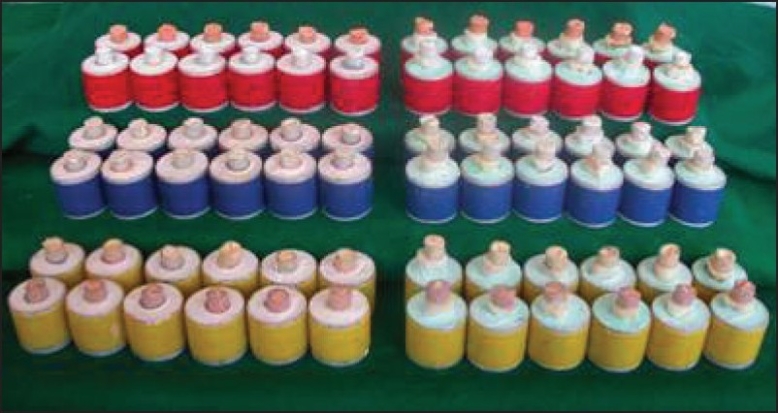
Total no. of Specimens

Seventy-two specimens were mounted on aluminum cylinders (20 mm in height) [Figure 1]. The cylinders were filled with plaster of Paris, and the specimens were placed on the top surface of the plaster rings.

Standardized preparations of flat dentin surfaces were obtained from extracted human maxillary and mandibular molars. A low-speed diamond disk saw with water coolant was used to slice off the occlusal 2 mm of the teeth, leaving a flat dentinal surface surrounded by a thin rim of enamel. This section of tooth, which included the roots, was embedded in plaster of Paris, face up within the plaster ring. The smear layer on dentin surface was removed by 17% Ethylene diamino tetra acetic acid (EDTA), 5.5% sodium hypochlorite (NaOCl). Finally, the dentin surface was dried with paper points.

Disks of gutta-percha were prepared by compacting a number of standardized cones of gutta-percha (Dentsply) [Figure 3]. The cones were softened by short immersion in a thermostat-controlled water bath (45±3°c) and compacted with a large plugger into a ring mold 10 mm in diameter and 2 mm high. During this operation, the ring was kept on a glass slab placed on the surface of a thermostat-controlled electric heater (45°C). Proper control of heating of gutta-percha is necessary to avoid physicochemical and molecular structural changes. After cooling, the gutta-percha disks were embedded in plaster of Paris at the surface of plaster ring. After the plaster set, the surface of pellet was slightly polished on wet waterproof polishing paper. Three strokes in two perpendicular directions on each grade served to standardize the surface preparation of each pellet.

**Figure 2:**
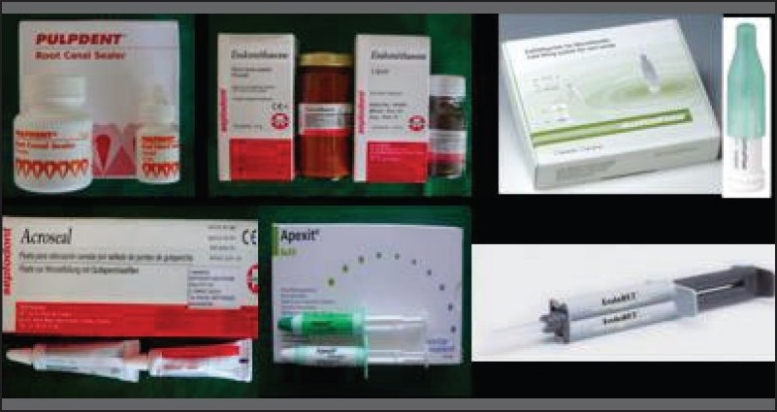
Materials used in the study

Five millimeter long sections of polyethylene tubing with ⅛-inch internal diameter and ¼-inch external diameter were filled with freshly mixed sealer [Figure 2] and carefully placed with one open side contacting the gutta-percha. Bonding to dentin surface was done similarly, with the dentin surface perpendicular to the tubing. The sealers were poured into the tubes that were held in contact with the gutta-percha disks and to the cut dentin surfaces, taking care to let the material flow to the substrate and avoid entrapping air bubbles [Figure 4].

**Figure 3:**
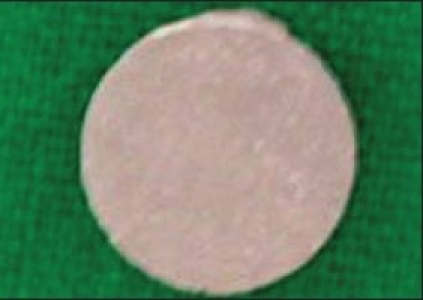
Guttapercha disk prepared

**Figure 4:**
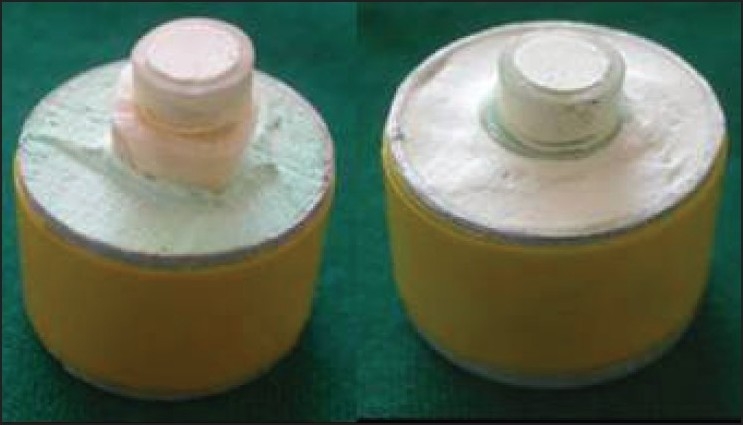
Materials placed

The specimens were randomly divided into two main experimental groups, with three subgroups under each main experimental group.

### Experimental groups

#### Group I: Thirty-six freshly extracted human molar specimens

Subgroup 1: Twelve specimens — zinc oxide eugenol-based sealers

Subgroup 1a: Six specimens — Pulpdent root canal sealer (Pulpdent)

Subgroup 1b: Six specimens — endomethasone sealer (Septodent)

Subgroup 2: Twelve specimens — calcium hydroxide–based sealers

Subgroup 2a: Six specimens — Acroseal sealer (Septodent)

Subgroup 2b: Six specimens — Apexit sealer (Ivoclar Vivadent)

Subgroup 3: Twelve specimens — resin-based sealers

Subgroup 3a: Six specimens — Guttaflow sealer (Coltene whaledent)

Subgroup 3b: Six specimens — EndoRez sealer (Ultradent)

The prepared sections of polyethylene tubing were filled with freshly mixed sealer and carefully placed with one open side contacting the cut dentin surface, perpendicular to its surface. The prepared specimens were stored in an incubator at 37°C for 24 hours.

#### Group II: Thirty-six gutta-percha disks embedded in plaster of Paris specimens

Subgroup 1: Twelve specimens — zinc oxide eugenol sealers

Subgroup 1a: Six specimens — Pulpdent root canal sealer (Pulpdent)

Subgroup 1b: Six specimens — endomethasone sealer (Septodent)

Subgroup 2: Twelve specimens — calcium hydroxide-based sealers

Subgroup 2a: Six specimens — Acroseal sealer (Septodent)

Subgroup 2b: Six specimens — Apexit sealer (Ivoclar Vivadent)

Subgroup 3: Twelve specimens — resin-based sealers

Subgroup 3a: Six specimens — Guttaflow sealer (Coltene whaledent)

Subgroup 3b: Six specimens — EndoRez sealer (Ultradent)

The prepared sections of polyethylene tubing were filled with freshly mixed sealer and carefully placed with one open side contacting the gutta-percha disk, perpendicular to its surface.

The surface area of contact was equal for all specimens (7.92 mm^2^).

All specimens were transferred to an incubator at 37°C (relative humidity, 100°) for 24 hours. The specimens were then mounted onto a metallic jig, and bond strength was tested using an Instron machine, Model 1011. The force required to break the bonds between the sealer and gutta-percha and between the sealer and dentin was recorded on the machine in newtons.

The samples were subjected to a shear load at a crosshead speed of 1 mm/min. The shear bond strength was calculated in megapascals (MPa), as this is an internationally accepted unit, by dividing the maximum tensile load at failure (in newtons) by the cross-sectional area of the bond. The results obtained were tabulated and statistically analyzed using Mann-Whitney *U* test [Table 1].

**Table 1:** Mean shear bond strength values and standard deviation in megapascals for Mann-Whitney *U* test

Material	Group	N	Mean	Std. deviation	Z
Pulpdent	GP	6	.9333	.08165	3.03500
Endomethasone		6	1.2500	.05477	*P*= .002 hs
Pulpdent	Dentin	6	.3000	.08944	2.93900
Endomethasone		6	.5500	.05477	*P*= .003 hs

hs: highly significant; *P*: probability

## RESULTS

When the data obtained was converted to megapascals and subjected to statistical analysis by Mann-Whitney *U* test, the following results were obtained [Figure 6]:

1. Subgroups 1a and 1b, Pulpdent and endomethasone, showed high statistically significant results when comparing the bond strength values of gutta-percha and dentin.
2. Subgroup 2a, Acroseal, showed no statistically significant difference in mean bond strength values between gutta-percha and dentin.
3. Subgroup 2b, Apexit (calcium hydroxide–based sealer), showed statistically significant difference in mean bond strength values between gutta-percha and dentin.
4. Subgroups 3a and 3b, Guttaflow and EndoRez (resin-based sealers), showed no significant difference in mean bond strength values between gutta-percha and dentin.

**Figure 5:**
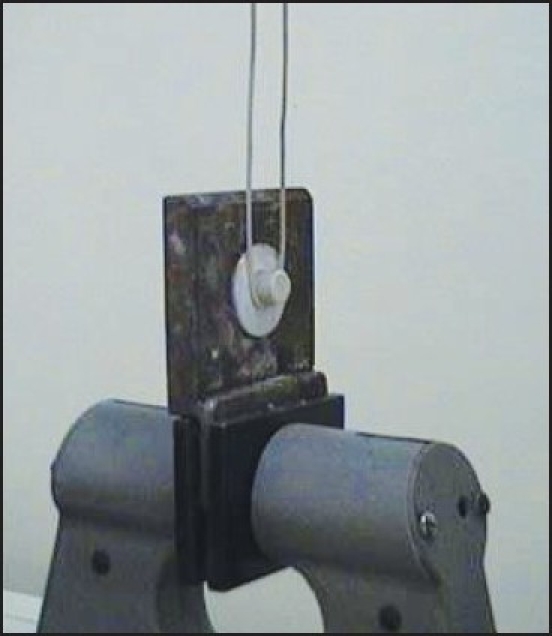
Specimen mounted

**Figure 6:**
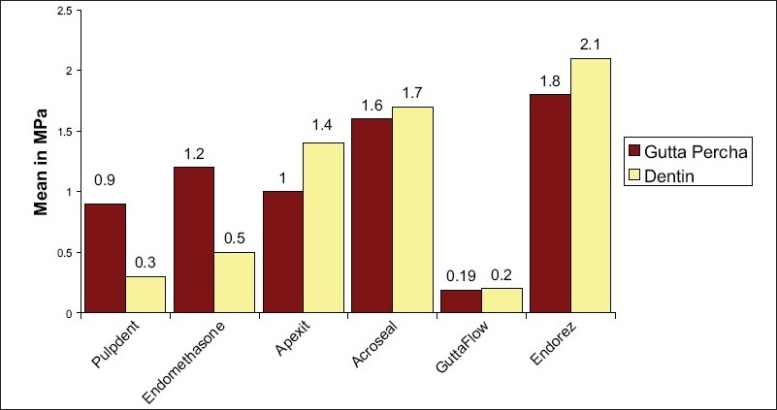
Mean values obtained for statistical analysis

### When inter-group comparison was done by Mann-Whitney *U* test –

1. Pulpdent *vs.* endomethasone showed high statistically significant difference in mean bond strength values to both gutta-percha and dentin [Table 2].

**Table 2:** Inter-group comparison was done by Mann-Whitney *U* test to compare between subgroups 1a and 1b, Pulpdent *vs.* endomethasone

Material	Group	N	Mean	Std. deviation	Z
Acroseal	GP	6	1.6333	.15055	1.72000
Apexit		6	1.4000	.24495	*P*= .086 ns
Acroseal	Dentin	6	1.7667	.10328	2.96100
Apexit		6	1.0000	.21909	*P*= .003 hs

hs: highly significant; ns: not significant; *P*: probability
2. Acroseal *vs.* Apexit showed no statistically significant difference in mean bond strength values to gutta-percha but showed high statistically significant difference in mean bond strength values to dentin [Table 3].

**Table 3:** Inter-group comparison was done by Mann-Whitney *U* test to compare between subgroups 2a and 2b, Acroseal *vs.* Apexit

Material	Group	N	Mean	Std. deviation	Z
Pulpdent	GP	6	.9333	.08165	3.00600
	Dentin	6	.3000	.08944	*P*= .003 hs
Endomethasone	GP	6	1.2500	.05477	2.96600
	Dentin	6	.5500	.05477	*P*= .003 hs
Acroseal	GP	6	1.6333	.15055	1.34400
	Dentin	6	1.7667	.10328	*P*= .179 ns
Apexit	GP	6	1.4000	.24495	2.45400
	Dentin	6	1.0000	.21909	*P*= .014 sig
Guttaflow	GP	6	.1833	.01033	.49600
	Dentin	6	.1967	.03882	*P*= .62 ns
Endorez	GP	6	1.8000	.00000	1.04900
	Dentin	6	2.1000	.30984	*P*= .294 ns

hs: highly significant; MPa: megapascal; sig: significant; ns: not significant
3. Guttaflow *vs.* EndoRez showed high statistically significant difference in mean bond strength values to both gutta-percha and dentin [Table 4].

**Table 4:** Inter-group comparison was done by Mann-Whitney *U* test to compare between subgroups 3a and 3b, Guttaflow *vs.* EndoRez

Material	Group	N	Mean	Std. deviation	Z
Guttaflow	GP	6	.1833	.01033	3.14600
Endorez		6	1.8000	.00000	*P*= .002 hs
Guttaflow	Dentin	6	.1967	.03882	2.96100
Endorez		6	2.1000	.30984	*P*= .003 hs

hs: highly significant

### When inter-group comparison of subgroups was done by Mann-Whitney *U* test –

1. Zinc oxide eugenol-based sealers *vs.* calcium hydroxide–based sealers showed very high statistically significant difference in mean bond strength values to both gutta-percha and dentin [Table 5].

**Table 5:** Final comparison was done by Mann-Whitney *U* test to compare subgroups 1a and 1b with subgroups 2a and 2b

Material	Group	N	Mean	Std. deviation	Z
1a - 1b	GP	12	1.0917	.17816	3.45900
2a - 2b		12	1.5167	.22896	*P*=.001 vhs
1a - 1b	Dentin	12	.4250	.14848	4.18100
2a - 2b		12	1.3833	.43240	*P*=.001 vhs

vhs: very highly significant
2. Zinc oxide eugenol-based sealers *vs.* resin-based sealers showed no statistically significant difference in mean bond strength values to both gutta-percha and dentin [Table 6].

**Table 6:** Final comparison was done by Mann-Whitney *U* test to compare subgroups 1a and 1b with subgroups 3a and 3b

Material	Group	N	Mean	Std. deviation	Z
1a - 1b	GP	12	1.0917	.17816	.00000
3a - 3b		12	.9917	.84431	*P*=1 ns
1a - 1b	Dentin	12	.4250	.14848	.34800
3a - 3b		12	1.1483	1.01603	*P*=.728 ns

ns: not significant; Z: Mann-Whitney U test

The present study indicates that the non–zinc oxide eugenol sealers showed better bond strength to dentin and gutta-percha when compared to zinc oxide eugenol sealers.

## DISCUSSION

An ideal endodontic sealer should adhere firmly both to dentin and gutta-percha.[2] The stability of a filling depends upon the bond of the sealer to gutta-percha and dentin.[3] The sealers vary markedly in their adhesion to dentin and gutta-percha.

There is a wide variation in the sealing capacity of different endodontic materials, and achieving an adequate apical seal is an important goal in endodontics.[4] When evaluating a new root canal filling material, analysis of its sealing ability is therefore very important.[5]

In this study, an attempt was made to duplicate more closely clinical condition in which only a thin layer of sealer is present between dentin and gutta-percha[6] where the model permitted calculation of combined bond strength of sealers to gutta-percha and dentin. It could not disclose the exact value of bond to each substrate, although this information may be sufficient at the clinical level. The gutta-percha used in the present study was made into a dimension of 10 mm diameter and 2 mm thickness, and the dentin substrate was 2 mm coronal cut molar teeth.

It is often stated that leakage may be influenced by the ability of root canal sealer to bond to dentinal wall or at least to maintain permanent contact with tooth structure. Adhesion of root canal fillings on dentinal walls seems to be advantageous for two main reasons.[7]

In a static situation, it should eliminate any space that allows the percolation of fluids between the filling and the wall.[6] In a dynamic situation, it is needed to resist dislodgment of the filling during subsequent manipulation. The adhesion depends on a multitude of interacting factors, including surface energy of the adherent (dentin or gutta-percha), the surface tension of the adhesive (sealer), and wet surface and cleanliness of adherent surface.[8]

Results of the present study showed that the non–zinc oxide eugenol sealers, i.e., calcium hydroxide– and resin-based sealers, had significantly better bond strength values when compared to zinc oxide eugenol sealers.

However, zinc oxide eugenol sealers, which were used as control, showed higher bond strength to gutta-percha in comparison to dentin. This can be attributed to the setting reaction of zinc oxide eugenol mixtures, which is a chelation reaction occurring with the zinc ion of the zinc oxide. This reaction may also occur with zinc oxide phase of gutta-percha, which ranges from 50% to 70% according to manufacturers, as well as with the calcium of the mineral phase of dentin.[9]

Data from our study showed that when the shear bond strength was compared among zinc oxide eugenol and non–zinc oxide eugenol sealers, statistical difference was seen for bond to gutta-percha and dentin. This is because of the fact that different sealers show different bond strengths to gutta-percha and dentin. This is in accordance with the study done by Saleh IM *et al.*,[8] that different sealers showed different adhesion to dentin and gutta-percha.

The bond strength of recently introduced silicone-based sealer has not been previously reported. In the present study, recently introduced Guttaflow, which is a polydimethyl siloxane–based resin; and EndoRez, which is a dimethacrylate urethane–based resin sealer, have been used for evaluation of sealing ability.

Resin-based root canal filling material provides a better seal but still may have difficulties because of polymerization shrinkage.[10] Another difficulty is the accessibility of adhesive systems and curing light into deeper parts of the root canal system. Moreover, the removal of the resin is difficult or impossible in curved root canals.[11] The manufacturer claims a better seal and good adaptability because of the increased flow ability. One of the possible explanations for better results of resin-based sealers compared to zinc oxide eugenol sealers and calcium hydroxide sealers, to dentin is the fact that they penetrate more into the dentinal tubules and get adapted to the dentinal walls; and this also creates micromechanical interlocking between dentin collagen and resin and thus helps to form a hybrid layer. This is in accordance with the study done by Sevimay S. *et al.*[12]

In the present study, resin-based sealers showed better shear bond strength values compared to the zinc oxide eugenol–based sealers.

In the present study, the shear bond strengths of eugenol-based (zinc oxide eugenol) sealers and non–zinc oxide eugenol (calcium hydroxide– and resin-based) endodontic sealers were compared. Results showed that there was significant difference in bond strength between zinc oxide eugenol and non–zinc oxide eugenol sealers to gutta-percha and dentin. Zinc oxide eugenol–based sealers showed better adhesion to gutta-percha, while calcium hydroxide– and resin-based sealers showed better adhesion to dentin. This is in accordance with the study done by Najar AL *et al.*[13]

The explanation for zinc oxide eugenol sealers to have high bond strength to gutta-percha and low bond strength to dentin is that eugenol can react with zinc oxide in gutta-percha to create a chelate bond because the two materials share common ingredients and because eugenol in excess may soften gutta-percha, increasing the sealer–gutta-percha interface.[9]

Adhesive strength is only one aspect of root canal sealers. Further investigation of various aspects of root canal sealers is necessary. Which sealers seal better in the presence, as well as the absence, of smear layer is one specific area that needs further evaluation. In addition, studies are needed regarding which sealer works best in specific situations such as open apices, apical deltas, ledged canals, and with specific obturation technique. The present evaluation examined only one aspect of the question of which sealer is best in terms of adhesion to dentin and gutta-percha. Further in *vivo* and in *vitro* investigations are recommended for a conclusive report on newer resin-based sealers.

## CONCLUSION

According to the methodology employed in the present study, it can be concluded that —

1. The shear bond strength of resin-based sealers (Guttaflow and EndoRez) showed the best adhesion to gutta-percha and dentin.
2. The greatest amount of adhesion to dentin was achieved by the resin-based sealer EndoRez.
3. The shear bond strength of zinc oxide eugenol–based sealers (Pulpdent and endomethasone) showed better adhesion to gutta-percha.
4. The shear bond strength of non–zinc oxide eugenol–based sealers (calcium hydroxide–based sealers Apexit and Acroseal and resin-based sealers Guttaflow and EndoRez) showed better adhesion to dentin.
5. The present study was an in *vitro* evaluation, where the sealer was in contact with the dentin surface and gutta-percha surface. Clinically, factors such as setting time, area of contact, mixing time, setting method, and temperature may modify the bond strength.
